# Dual Acting Neuraminidase Inhibitors Open New Opportunities to Disrupt the Lethal Synergism between *Streptococcus pneumoniae* and Influenza Virus

**DOI:** 10.3389/fmicb.2016.00357

**Published:** 2016-03-21

**Authors:** Elisabeth Walther, Zhongli Xu, Martina Richter, Johannes Kirchmair, Ulrike Grienke, Judith M. Rollinger, Andi Krumbholz, Hans P. Saluz, Wolfgang Pfister, Andreas Sauerbrei, Michaela Schmidtke

**Affiliations:** ^1^Department of Virology and Antiviral Therapy, Jena University HospitalJena, Germany; ^2^Center for Bioinformatics, University of HamburgHamburg, Germany; ^3^Department of Pharmacognosy, University of ViennaVienna, Austria; ^4^Institute for Infection Medicine, Christian-Albrecht University of Kiel–University Medical Center Schleswig-Holstein, Campus KielKiel, Germany; ^5^Leibniz Institute for Natural Product Research and Infection Biology – Hans Knöll InstituteJena, Germany; ^6^Department of Medical Microbiology, Jena University HospitalJena, Germany

**Keywords:** pneumococci, secondary pneumococcal pneumonia, co-pathogenesis, pandemic influenza, enzyme inhibition, antibacterial, antiviral, microbial communication

## Abstract

Secondary infections with *Streptococcus pneumoniae* cause severe pneumonia and enhance lethality during influenza epidemics and pandemics. Structural and functional similarities with viral neuraminidase (NA) suggest that the highly prevalent pneumococcal NAs, NanA and NanB, might contribute to this lethal synergism by supporting viral replication and that dual acting NA inhibitors (NAIs) will disrupt it. To verify this hypothesis, NanA and NanB were expressed in *E. coli*. After confirming their activity in enzyme assays, *in vitro* models with influenza virus A/Jena/8178/09 (Jena/8178) and the recombinant NanA or NanB (rNanA and rNanB) were established in A549 and MDCK cells to mimic the role of these pneumococcal NAs during co-infection. Studies on the influence of both NAs on viral receptor expression, spread, and yield revealed a distinct effect of NanA and NanB on viral replication in these *in vitro* models. Both enzymes were able to support Jena/8178 replication at certain concentrations. This synergism was disrupted by the NAIs oseltamivir, DANA, katsumadain A, and artocarpin exerting an inhibitory effect on viral NA and NanA. Interestingly, katsumadain A and artocarpin inhibited rNanA and rNanB similarly. Zanamivir did not show activity. These results demonstrate a key role of pneumococcal NAs in the lethal synergism with influenza viruses and reveal opportunities for its effective disruption.

## Introduction

The highly variable influenza A viruses evolve rapidly and cause epidemics and pandemics of acute respiratory disease that are often characterized by high morbidity and mortality in humans (Webster and Govorkova, 2014). Secondary *Streptococcus* (*S.*) *pneumoniae* infections increase the severity and lethality in influenza virus-infected humans based on co-pathogenesis of both pathogens, also called lethal synergism as reviewed recently (McCullers, 2014). “Despite advances in medical care and numerous new antibiotics, the case fatality rate for complicated bacterial pneumonia has not decreased appreciably since the introduction of penicillin" (McCullers and English, 2008). However, treatment with neuraminidase inhibitors (NAI) reduces the risk of lower respiratory tract complications and mortality (Muthuri et al., 2014; Dobson et al., 2015).

Due to the grave medical and economic impact of this lethal synergism between influenza A viruses and *S. pneumoniae*, the underlying viral and bacterial factors promoting it are under intensive investigation. The lethal synergism requires influenza virus infection prior to bacterial exposure (McCullers and Rehg, 2002). Therefore, several viral factors, in particular the viral neuraminidase (NA) contributing to secondary infection with *S. pneumoniae* by providing attachment receptors and nutrients to the bacteria, were thoroughly investigated (McCullers and Bartmess, 2003; Peltola and McCullers, 2004; McCullers, 2014; Siegel et al., 2014). In mice, NAI abolished the viral support for bacterial adherence and improved the outcome of *S. pneumoniae* induced secondary pneumonia (McCullers and Bartmess, 2003; McCullers, 2004; Peltola and McCullers, 2004; Tanaka et al., 2014).

In contrast, the role of pneumococcal virulence factors was less studied until now. Rebound of virus titers was detected after co-infection with *S. pneumoniae in vivo* due to an enhanced viral release from infected cells (McCullers and Rehg, 2002; Smith et al., 2013). It implied the strengthened NA activity from pneumococci promoted the virus release. In addition, rescue of influenza virus release by *S. pneumoniae in vitro* from the inhibition of NAI zanamivir (Nishikawa et al., 2012) further indicated pneumococcal NAs empowered the observed virus titers rebound *in vivo*.

*Streptococcus pneumoniae* expresses three distinct NAs: NanA, NanB, and NanC (Pettigrew et al., 2006; Xu et al., 2011; Walther et al., 2015). The most active and highly expressed NanA (Berry et al., 1996; Manco et al., 2006) was detected in all *S. pneumoniae* strains and has a conserved catalytic site (King et al., 2005; Pettigrew et al., 2006; Walther et al., 2015). NanA hydrolyzes α2,3-, α2,6-, and α2,8-sialyllactose to release *N*-acetyl-neuraminic acid (Neu5Ac; Xu et al., 2011). It supports colonization and sepsis *in vivo* (Orihuela et al., 2004; Manco et al., 2006; Song et al., 2008). Its impact on biofilm formation is in discussion (King et al., 2004; Oggioni et al., 2006; Parker et al., 2009; Trappetti et al., 2009; Walther et al., 2015). NanB, detected in most but not all *S. pneumoniae* strains (Pettigrew et al., 2006; Walther et al., 2015), represents a *trans*-sialidase transferring 2,7-anhydro-Neu5Ac from α2,3-sialosides to other glycoconjugates (Gut et al., 2008; Xu et al., 2008, 2011) and contributes to deglycosylation of sialic acids (SA) *in vitro* (Burnaugh et al., 2008) and *in vivo* (Tong et al., 2001; Siegel et al., 2014). NanB deficient strains are unable to colonize the nasopharynx or to cause sepsis (Manco et al., 2006). NanB is highly expressed in biofilm (Manco et al., 2006; Oggioni et al., 2006). Its different substrate specificity (Gut et al., 2008; Xu et al., 2011) together with slightly different pH optima (pH 5.5-6.5 for NanA, pH 5.0–5.5 for NanB; Hayre et al., 2012) suggests a distinct role of NanB in bacterial pathogenicity and in the synergism between influenza viruses and *S. pneumoniae*. In contrast to NanA and NanB, NanC is rarely expressed (Pettigrew et al., 2006; Walther et al., 2015). Interestingly, it was suggested to regulate the activity of NanA *via* producing 2-deoxy-2,3-didehydro-*N*-acetylneuraminic acid (DANA), a known NAI (Xu et al., 2011; Owen et al., 2015).

Despite the distinct quaternary structures of viral and pneumococcal NAs (viral NA as tetramer and bacterial NAs as monomer), their active sites show close similarities (von Grafenstein et al., 2015), suggesting that *S. pneumoniae* could be targeted by NAIs. However, the viral NAI zanamivir does not bind efficiently to the active site of NanA and NanB because the residues involved in key interactions (e.g., Glu119 and Glu227 in the viral NA: N2 numbering) are not conserved in *S. pneumoniae* NAs (Gut et al., 2011). In contrast, oseltamivir competitively inhibits NanA (Gut et al., 2011; Walther et al., 2015). Two natural compounds, katsumadain A and the isoprenylated flavone artocarpin, also inhibited pneumococcal NA activity (Richter et al., 2015; Walther et al., 2015). Hence, the dual acting NAIs (active against both viral and bacterial NA) might have the potential to combat the lethal synergism between influenza virus and secondary pneumococcal infection by targeting the interaction between both pathogens.

The present study aims (i) to verify the impact of pneumococcal NanA and NanB on influenza virus replication and (ii) to evaluate the potential of dual acting NAI *via in vitro* influenza virus-*S. pneumoniae* co-infection model. We expressed NanA or NanB in *E. coli* to achieve recombinant NAs (rNanA or rNanB) and established cell-based models with the A(H1N1)pdm09 isolate A/Jena/8178/09 (Jena/8178) and the recombinant NAs to mimic the co-infection with *S. pneumoniae.* We used the models to evaluate the effect of pneumococcal NAs on the spread and yield of the virus, and the availability of viral receptors. Furthermore, the efficacy of NAIs was studied under experimental conditions mimicking the lethal synergism of influenza virus and *S. pneumoniae*.

## Materials and Methods

### Compounds

Oseltamivir carboxylate (GS4071; GlaxoSmithKline, Uxbridge, UK), zanamivir (GG167; F. Hoffmann-La Roche AG, Basel, Switzerland), and *N*-acetyl-2,3-dehydro-2-deoxyneuraminic acid (DANA; Sigma–Aldrich GmbH, Taufkirchen, Germany) were dissolved in bi-distillated water. Artocarpin (Quality Phytochemicals LLC, East Brunswick, NJ, USA) and katsumadain A, previously isolated from the seeds of *Alpinia katsumadai* Hayata (Grienke et al., 2010) were dissolved in DMSO. Except for DANA (stored at –20°C), compound stocks (10 mM) were stored at 4°C until use.

### Cells

Human lung carcinoma cells (A549; Institute of Molecular Virology, University of Münster, Germany) were propagated in Dulbecco’s Modified Eagle Medium (DMEM; Lonza Group Ltd, Basel, Switzerland) supplemented with 10% fetal calve serum (PAA Laboratories GmbH, Cölbe, Germany). Madin–Darby canine kidney (MDCK) cells (Friedrich-Loeﬄer Institute, Riems, Germany) were grown in Eagle’s minimum essential medium (EMEM; Lonza Group Ltd) supplemented with 10% fetal bovine serum (Sigma–Aldrich GmbH), and 2 mM L-glutamine (Lonza Group Ltd), 10% non-essential amino acids (Lonza Group Ltd), 1 mM sodium pyruvate (Lonza Group Ltd.). Cells were maintained in a humidified incubator at 37°C with 5% CO_2_.

Test medium used for virus propagation and *in vitro* studies with virus was serum-free but contained 0.25 μg/mL (A549 cells) or 2 μg/mL (MDCK cells) trypsin (Sigma–Aldrich GmbH) and 1.3% sodium bicarbonate (Lonza Group Ltd.).

### Virus

The stock of the A(H1N1)pdm09 influenza virus isolate A/Jena/8178/09 (Jena/8178) was prepared in MDCK cells, aliquoted, and stored at –80°C until use.

### Expression of rNanA and rNanB

The expression and purification of *nanA* of *S. pneumoniae* DSM20566 has been described previously (Walther et al., 2015). The *nanB* from the same strain was amplified with the primers: nanB_NcoI_fw (5′-GGAAAACCATGGATAAAAGAGG-3′) and nanB_XhoI_rv (5′-TTCTCTCGAGTTTTGTTAAATC-3′). After the double-digestion with *Nco*I and *Xho*I, the ∼2.1 kb PCR product was cloned into *E. coli* expression vector pET-28a. The obtained plasmid pTNB20566 encodes a C-terminal 6xHis-tagged NanB in size of 699 amino acid residues. Expression and purification of NanB was done as described for NanA (Walther et al., 2015).

### Analysis of Pneumococcal NA Activity

Activity of 10-fold dilutions (1:1,000 to 1:100,000) of rNanA and rNanB was determined with a fluorescence-based NA assay (FL assay) using the substrate 2-(4-methylumbelliferyl)-α-D-*N*-acetylneuraminic acid sodium salt hydrate (MUNANA, Sigma–Aldrich GmbH) as published (Richter et al., 2015). The activity of rNanB was additionally tested at pH 5. Relative FL units (RFU) were read using the microplate reader FLUOstar Omega (BMG Labtech GmbH, Ortenberg, Germany) and converted to 4-MU concentration (Sigma–Aldrich GmbH) according to the 4-MU standard curve (Supplementary Figure 1). Velocity (μM/min) and *K*_m_ value (μM) for rNanA and rNanB were determined using eight MUNANA concentrations (3.13 to 400 μM) at pH 6.5. After 5 min the reaction was stopped, RFU were measured and converted to 4-MU concentrations according to the 4-MU standard curve. The Enzyme Kinetics Module of SigmaPlot 12.0 (Systat Software, San Jose, CA, USA) was applied to fit the data to the Michaelis–Menten equation using non-linear regression.

Neuraminidase activity of both NAs was further confirmed based on hemagglutination of NA-treated human erythrocytes in the presence of peanut lectin (HA assay) as described recently (Richter et al., 2015; Walther et al., 2015).

### NA Inhibition

The NAI susceptibility of rNanA and rNanB was evaluated with FL assay (rNanA and rNanB diluted 1:10,000 in FL buffer; pH 6.5) and HA assay as published recently (Richter et al., 2015) with slight modification of the HA assay. For stability reasons, rNanA and rNanB were diluted 1:5,000 in phosphate buffered saline (PBS) containing 100 μg/mL bovine serum albumin and stored at –20°C until use. The incubation period of the recombinant proteins with the human erythrocytes was only 1 h. At least three individual experiments were performed.

NAI susceptibility of Jena/8178 NA was proved in the FL assay.

### Effects of Recombinant NAs on Viral Replication *In Vitro*

Confluent MDCK and A549 cells were mock-infected with test medium or infected with Jena/8178 (0.1 TCID_50_/cell) for 1 h (37°C; 5% CO_2_). After three washing steps with test medium, test medium or various rNanA or rNanB dilutions (1:100 to 1:1,000,000) were added. After 48 h at 37°C the supernatant was taken and stored at –20°C until plaque titer determination (virus yield analysis). The remaining cells were fixed with ice-cold methanol for 20 min and stored after washing with PBS in PBS at 4°C until immunocytochemical staining (virus spread analysis).

### Virus Yield and Spread Analysis

To determine the virus yield in collected supernatants, plaque assays were conducted on confluent MDCK cells grown in 12-well tissue culture plates (Greiner Bio-One GmbH). Briefly, after virus adsorption (37°C; 1 h), inoculum was replaced by 1 mL test medium containing also 0.4% agarose and 50 mM magnesium chloride (VEB Laborchemikalien, Apolda, Germany). After 48 h of incubation at 37°C, cells were fixed and stained with a solution of 0.4% crystal violet in a mixture of formalin (3% v/v) and ethanol (1.67% v/v) in water overnight. Plaques were counted over a light box after removal of the agar overlay.

Virus spread was analyzed by detecting viral nucleoprotein with a monoclonal antibody (Acris GmbH, Hiddenhausen, Germany) by immunocytochemical staining according to the manufacturers protocol (DAKO, Hamburg, Germany; Bauer et al., 2007).

### Effects of Recombinant NAs on Inhibition of Virus Replication by NAI

The inhibitory effect of the NAI on Jena/8178 (inhibition of virus yield and spread) was determined in absence and presence of rNanA or rNanB in A549 cells. NA dilutions were used that (i) did not reduce the amount of SA α2,3-Galactose (Gal) and SA α2,6-Gal but, (ii) significantly increased virus yield and spread.

### Detection of Sialic Acid (SA) α2,3-Galactose (Gal) and SA α2,6-Gal

Confluent A549 or MDCK cells were incubated for 48 h at 37°C with 10-fold diluted NanA or NanB. After methanol fixation the effect of pneumococcal NAs on cell surface SA was investigated by immunocytochemical staining with a DIG glycan differentiation kit (F. Hoffmann-La Roche AG) as reported (Seidel et al., 2014). DIG-labeled *Sambucus nigra* agglutinin (SNA) or *Maackia amurensis* agglutinin (MAA) recognizes SA α2,6-Gal and SA α2,3-Gal, respectively. The red staining chromogenic solution of the DAKO REAL Detection System APAAP, Mouse (DAKO, Hamburg, Germany), was used for SNA and MAA detection.

### Molecular Modeling

Homology models of Jena/8178 and NanA and NanB of *S. pneumoniae* DSM20566 were generated with Swiss-Model (Biasini et al., 2014) using PDB 4b7q, 2vvz and 2jkb as templates, respectively. Oseltamivir and zanamivir were extracted from aligned co-crystallized structures (2hu0 and 4b7q) and inserted into the homology models. Residue numbering of the template structures was preserved. Graphics were rendered using PyMol version 0.99 (DeLano Scientific LLC, 2006).

### Statistical Analysis

Means and standard deviations were analyzed using Microsoft Excel 2010. Significant differences in the co-incubation assay were calculated in SPSS Statistics Version 22 with non-parametric Wilcoxon–Mann–Whitney test.

## Results

### Effects of NanA and NanB on Virus Replication

*Streptococcus pneumoniae* induced cytopathic effects in A549 and MDCK cells susceptible and permissive for Jena/8178, even at low multiplicities of infection (MOI; results not shown). Therefore, we added recombinant protein rNanA and rNanB at different concentrations to Jena/8178-infected A549 and MDCK cells at the time of viral infection to probe the role of pneumococcal NAs during the co-infection.

Both rNanA and rNanB were expressed in *E. coli* and their activity was confirmed by the FL assay at pH 5 (for rNanB only) and pH 6.5 (for both rNanA and rNanB). The *K*_m_ value of rNanA (39.70 ± 5.81 μM) differed at least by one order of magnitude to that of rNanB (684.03 ± 162.76 μM), indicating a much stronger affinity of rNanA to MUNANA under these assay conditions (Supplementary Figure 2). Activities of both recombinant *S. pneumoniae* NAs were further confirmed under more physiological conditions in a hemagglutination assay (HA assay; Richter et al., 2015). In this HA assay, the removal of terminal SA at the surface of erythrocytes by rNanA and rNanB allowed the hemagglutination *via* peanut lectin as the approval of enzyme activity (results not shown).

**FIGURE 1:**
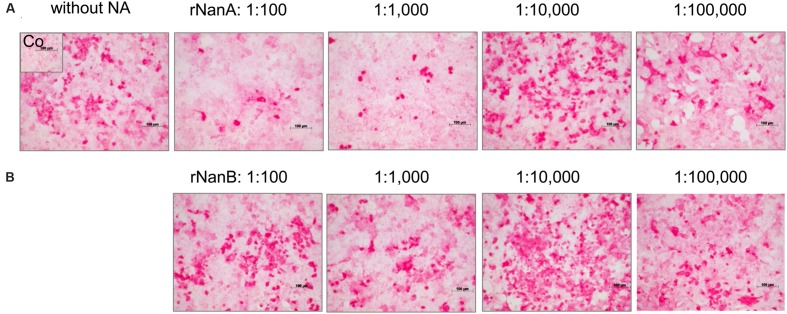
**Spread of A(H1N1)pdm09 strain A/Jena/8178/09 (Jena/8178) in the absence and the presence of different dilutions of recombinant NanA and NanB**. The effect of NanA **(A)** and NanB **(B)** on virus spread in A549 cells was analyzed by immunocytochemical staining of viral nucleoprotein (shown in red) 48 h after infection with Jena/8178 at MOI of 0.1 TCID_50_/cell.

Thereafter, we evaluated the effect of both *S. pneumoniae* NAs on Jena/8178 replication in A549 and MDCK cells. The applied MOI of 0.1 TCID_50_/cell resulted in only few infected cells during the first cycle of viral replication (Supplementary Figure 3). To study the influence of rNanA and rNanB on viral replication, different 10-fold enzyme dilutions were added at the time of virus inoculation. Forty-eight hours after infection, immunocytochemical staining was used to visualize the virus spread in NA-treated and untreated cells. In parallel, the virus yield of the supernatant was determined by plaque assay. Intriguingly, less diluted rNanA and rNanB (dilutions less than 1:1,000 and 1:100 for rNanA and for rNanB, respectively) hampered the spread of the virus in A549 cells (**Figures 1A,B**). In contrast, higher dilutions of rNAs (dilutions more than 1:10,000 and 1:1,000 for rNanA and for rNanB, respectively) promoted virus spread. Similar results were obtained with MDCK cells (Supplementary Figure 4). The yield of virus under the same experiment conditions was in good agreement with these results. The addition of less diluted pneumococcal NA to Jena/8178-infected A549 cells led to a decrease yield of virus, and *vice versa* (**Table 1**). The 1:1,000,000 dilutions of both NAs had no more effect on virus growth. These results clearly demonstrate that at certain concentrations both NanA and NanB are capable of enhancing the replication of influenza virus.

**Table 1:** Effect of recombinant NanA (rNanA) and NanB (rNanB) on virus yield in A549 cells.

Dilution of NAs	Percentage of virus yield in the presence of	
	
	rNanA^a^	rNanB^a^
1:100	2.8 ± 4.6	15.5 ± 4.5
1:1,000	12.2 ± 5.7	204.8 ± 77.5
1:10,000	234.4 ± 70.5	202.5 ± 107.3
1:100,000	171.7 ± 37.1	186.1 ± 34.5
1:1,000,000	103.2 ± 31.7	114.8 ± 47.4

*^a^The plaque titer of influenza virus A/Jena/8178/09 reached in A549 cells (48 h after infection) was determined in MDCK cells. Plaque titer without pneumococcal neuraminidases was set to 100% virus yield and used to calculate the percentage of virus yield in presence of rNanA and rNanB. Mean and standard deviations of at least two assays with two replicates are shown*.

### NanA and NanB Diminish Virus Receptor Availability

**FIGURE 2:**
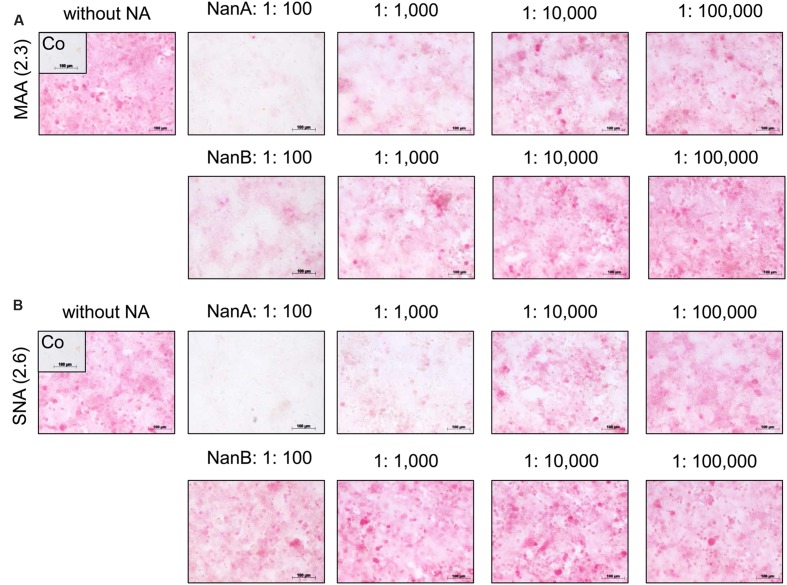
**Influence of recombinant NanA and NanB on expression of SA on the surface of A549 cells**. Cells were treated with different neuraminidase dilutions for 48 h. The lectins MAA **(A)** and SNA **(B)** were used to detect SAα2-3Gal and SAα2-6Gal, respectively, by immunocytochemical staining. Control (Co) was stained without using lectins.

The reduced viral spread and yield in the presence of high amounts of pneumococcal NAs suggests a removal of viral receptors by these enzymes. SA linked *via* α2,3 or α2,6 to galactose (SA α2,3-Gal and SA α2,6-Gal) function as receptors for influenza viruses and facilitate their attachment to host cells, the initial step in the viral replication cycle (Nicholls et al., 2013). To investigate the influence of rNanA and rNanB on virus receptors, we used the lectins MAA and SNA, respectively, to detect SAα2-3Gal and SAα2-6Gal on the cell surface. Both receptors of influenza A virus could be identified on A549 (**Figures 2A,B**) and MDCK cells (Supplementary Figure 5), which is consistent with previously published data (Kumari et al., 2007; Bauer et al., 2012; Seidel et al., 2014). SA α2,3-Gal and SA α2,6-Gal were strikingly reduced by rNanA at 1:100 and 1:1,000 dilutions when compared to the untreated cell control (**Figures 2A,B**). A decrease of SA α2,3 was induced by 1:100 and 1:1,000 diluted rNanB, whereas no obvious changes at SAα2,6 levels were observed (**Figures 2A,B**). No apparent reduction of the receptors was observed after the NA treatment with dilutions larger than 1:10,000. Similar effects were observed in MDCK cells (Supplementary Figure 5).

### Katsumadain A and Artocarpin Target NanA and NanB

Recently, we described a HA assay with highly robust readout for comparing the efficiency of NAIs against viral and bacterial NAs of the protein precipitate of *S. pneumoniae* DSM20566 (Richter et al., 2015). *S. pneumoniae* DSM20566 expresses NanA and NanB (Walther et al., 2015). Here we extended these studies and compared the NAI activity against rNanA and rNanB of *S. pneumoniae* DSM20566. In addition to oseltamivir, zanamivir and DANA (used as control NAI), the inhibitory potentials of katsumadain A and artocarpin were studied.

In the HA assay, oseltamivir, DANA, katsumadain A, and artocarpin but, not zanamivir inhibited rNanA (**Table 2**). Compared to rNanA, rNanB was approximately 10-times less susceptible to oseltamivir. Zanamivir and DANA were inactive. Interestingly, katsumadain A and artocarpin inhibited both NAs with similar efficiency. In contrast to rNanA, rNanB was not susceptible to artocarpin in the FL assay (**Table 2**). Apart from this discrepancy, the FL assay data confirmed the HA assay results. Due to the self-fluorescence of katsumadain A (Richter et al., 2015), the compound could not be tested in the FL assay.

**Table 2:** Inhibition of recombinant NanA (rNanA) and NanB (rNanB) by neuraminidase inhibitors (NAIs).

NAI	Inhibitory Concentration of NAIs in μM against			
	rNanA		rNanB	
	FL assay^a^	HA assay^b^	FL assay^a^	HA assay^b^
Oseltamivir	2.9 ± 1.0	3.2 ± 0.0	76.8 ± 27.4	31.6 ± 0.0
Zanamivir	Not active	Not active	Not active	Not active
DANA	17.7 ± 2.0	100.0 ± 0.0	Not active	Not active
Katsumadain A	Not evaluable^c^	3.2 ± 0.0	Not evaluable^c^	5.4 ± 4.0
Artocarpin	10.0 ± 6.3	10.0 ± 0.0	Not active	10.0 ± 0.0

*^a^Mean 50% inhibitory concentration (IC_*50*_) and standard deviation determined in FL assay in at least three independent assays. The maximum tested concentration was 100 μM. ^b^Mean minimal inhibitory concentration (MIC) and standard deviation determined in HA assay in at least three independent assays. The maximum tested concentration was 100 μM. ^c^Not evaluable due to self-fluorescence of the compound (Richter et al., 2015)*.

### Targeting the Interaction of *S. pneumoniae* NAs with Influenza Virus by NAI

**FIGURE 3:**
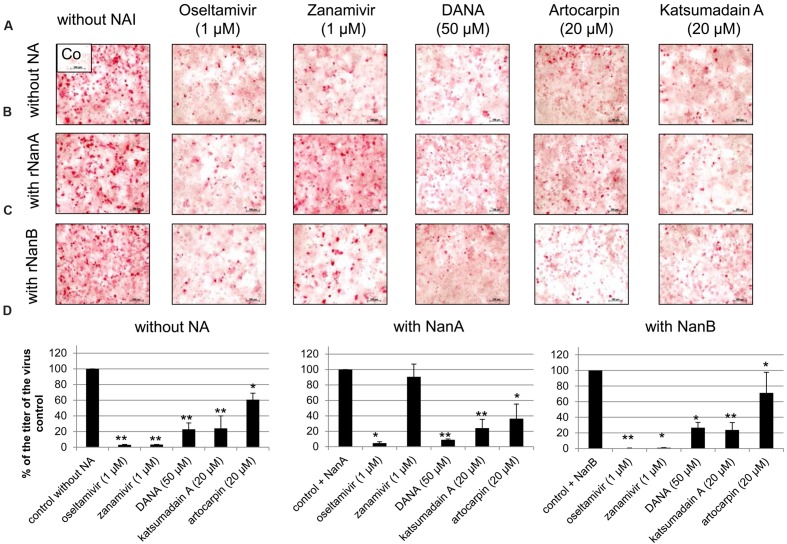
**Inhibition of replication of A(H1N1)pdm09 strain A/Jena/8178/09 (Jena/8178) by neuraminidase inhibitors (NAIs)**. A549 cells infected with Jena/8178 (MOI of 0.1 TCID_50_/cells) were treated with oseltamivir, zanamivir, DANA, artocarpin, and katsumadain A in absence of pneumococcal NAs **(A)**, presence of rNanA **(B)** or presence of rNanB **(C)**. Virus-infected cells were detected by immunocytochemical staining of viral nucleoprotein (shown in red) 48 h after infection to visualize the inhibitor effect on virus spread. To analyze the effect of NAIs on virus yield **(D)**, virus titers in pfu/mL were determined with plaque assay 48 h after infection. Virus control titer was set to 100% and inhibition of the control titer by NAIs in % was calculated. Experiments were performed at least three times, and one representative assay is exemplarily shown. Significant values were calculated with non-parametric Wilcoxon–Mann–Whitney test (^∗^*p* < 0.5, ^∗∗^*p* < 0.1).

A dilution of 1:10,000 of both pneumococcal NAs supported Jena/8178 replication (**Table 1** and **Figure 1**). Therefore, this dilution was selected to study the effect of NAIs on Jena/8178 spread and yield in the presence and absence of rNanA or rNanB. The NA of Jena/8178 has been proved to be susceptible to all tested NAIs (Supplementary Table 1). In the absence of pneumococcal NAs, NAIs reduced Jena/8178 spread and yield in A549 cells (**Figure 3**, Supplementary Table 2).

The antiviral effect of zanamivir was abolished in the presence of rNanA (**Figures 3B,D**, Supplementary Table 2) because rNanA was insensitive to zanamivir (**Table 2**) but able to cleave SA α2,3-Gal as well as SA α2,6-Gal like the viral NA (**Figure 2**). Thus, rNanA was fully capable to replace the function of viral NA when it was blocked by zanamivir. Both spread and yield of the virus were inhibited by oseltamivir (1 μM), DANA, katsumadain A, and artocarpin despite the presence of NanA. These NAIs block rNanA activity (**Table 2**). However, at lower concentration of 0.1 μM, the antiviral effect of oseltamivir was abrogated by rNanA (Supplementary Table 2).

Independently from their inhibitory activity against rNanB (**Table 2**), all tested NAIs were active in the presence of rNanB and reduced the spread and yield of virus (**Figures 3C,D**, Supplementary Table 2). Thus, the presence of rNanB did not affect NAI efficacy against Jena/8178. At first glance this was a surprising result. However, in contrast to viral NA, which predominantly cleaves SA α2,6-Gal, NanB only cleaves SA α2,3-Gal (Gut et al., 2008; Xu et al., 2008, 2011). Therefore, NanB cannot replace the function of the inhibited viral NA.

## Discussion

In the present study, we demonstrate the critical role of *S. pneumoniae* NanA and NanB for influenza virus replication in a newly established *in vitro* model. NAIs acting against viral and bacterial NAs were shown to disrupt the observed *S. pneumoniae* NA-based synergism in this model.

High amounts of rNanA and rNanB removed the SA from A549 and MDCK cell surfaces that are used as viral receptors thereby compromising Jena/8178 replication. Influenza virus A(H1N1)pdm09 isolates preferentially attach to SA α2,6-Gal (Stevens et al., 2006; Childs et al., 2009; Seidel et al., 2014). Therefore, removal of SA α2,6-Gal and/or SA α2,3-Gal by pneumococcal NAs (Xu et al., 2011) impairs or even blocks the replication of Jena/8178. Notably, removal of SA-containing receptors from respiratory epithelial cells is also the mechanism of action of the investigational antiviral drug DAS181 (Fludase; Nicholls et al., 2013). The obtained results help to explain why synergism only occurs “when the viral infection precedes bacterial exposure and no effect – or even protection – occurs when the order of challenge is reversed” (McCullers, 2014).

An opposed effect was observed in A549 cells infected with Jena/8178 when rNanA or rNanB dilutions were used that do not lead to a significant cleavage of SA from the receptors. Under these experimental conditions the pneumococcal NAs effectively enhanced the cleavage function for viral release, therefore promoted the viral replication. It demonstrated that both rNanA and rNanB contributed to the rebound of virus titers observed during the secondary pneumococcal infection *in vivo* (McCullers and Rehg, 2002; Smith et al., 2013). However, the effect will depend on the receptor specificity of a given virus.

**FIGURE 4:**
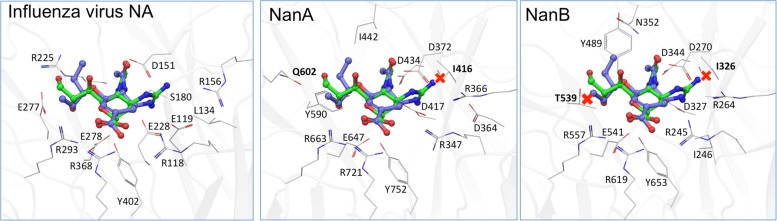
**Computational models of the binding of oseltamivir (carbons blue) and zanamivir (carbons green) to A/Jena/8178/09, NanA and NanB**. While both inhibitors fit well to the viral NA **(A)**, steric clashes (marked by a red X) of zanamivir with I416 in NanA **(B)** and the equivalent in NanB, I326, and T539 **(C)** are apparent. These are likely the cause for the observed inactivity of zanamivir on both bacterial NAs.

The distinct role of NanA and NanB in the co-pathogenesis of influenza virus and *S. pneumoniae* are also apparent from the data of inhibition assays with NAIs. As published for NanA (Hayre et al., 2012; Nishikawa et al., 2012; Richter et al., 2015; Walther et al., 2015) and confirmed by the enzyme inhibition assays in the present study, zanamivir is unable to inhibit the bacterial NAs activity till 100 μM. When co-incubating the virus, zanamivir and rNanA, “bacterial neuraminidases functioned as the predominant NA when viral NA was inhibited to promote the spread of infection” (Nishikawa et al., 2012). The same effect was observed for the low oseltamivir concentration (0.1 μM) that is ineffective against rNanA (Richter et al., 2015). In contrast, virus yield and spread remain reduced in the presence of NanB, leading to the conclusion that NanB cannot replace the NA function of Jena/8178 as NanA does. Unlike NanA, which can cleave both SA linkages, NanB has limited activity to cleave only SA α2,3-Gal (Gut et al., 2008; Xu et al., 2008, 2011). For this reason, NanB can only partially replace the cleavage function of Jena/8178 NA for an effective viral release and spread. In agreement with the fact that NanA is susceptible to oseltamivir, the addition of NanA did not rescue the viral spread. Due to the structural differences within the active sites of viral and pneumococcal NAs, the inhibition efficiency of oseltamivir toward NanA is markedly lower (Gut et al., 2011). We modeled the three NAs in question with oseltamivir and zanamivir bound (**Figure 4**). These models are in agreement with the collected *in vitro* data and are able to explain the observed inhibitory activity. It was found that steric clashes are the primary reason for inactivity on one or several of these structurally related enzymes.

In contrast to oseltamivir and DANA, katsumadain A and artocarpin were effective against NanA and NanB in the HA assay and inhibited the viral NA as well, which underlines our previous finding about the extended interaction of these natural compounds with NAs (Grienke et al., 2010; Kirchmair et al., 2011; Richter et al., 2015; Walther et al., 2015). They also inhibited viral replication. Moreover, artocarpin exerted antibacterial activity (Walther et al., 2015). Therefore, we suggest that its structure could provide the base for the search of compounds with stronger activity against both influenza viruses and *S. pneumoniae.*

In summary, our findings suggest distinct roles for NanA and NanB in the lethal synergism of A(H1N1)pdm09 strains and *S. pneumoniae*, as well as for influenza treatment with NAIs. It should be considered that their effect might depend on the virus strain due to the differences in the use of SAα2,6-Gal and/or SAα2,3-Gal modified receptors (Kumari et al., 2007). For example, the impact of NanB on avian or avian-like swine viruses that preferentially bind to SAα2,3-Gal might be much stronger (Nicholls et al., 2007; Bauer et al., 2012). The effect of viral NAIs that are unable to inhibit the pneumococcal NanA can be diminished. In consequence, only NAIs targeting viral and pneumococcal NanA as well NanB will help to prevent the lethal synergism.

## Author Contributions

EW, WP, HS, JR, and MS contributed to the conception or design of the work; EW (confirmed the NA activity as well as its inhibition in FL assays, established the *in vitro* model with A549 cells and analyzed SA expression, virus replication, and NAI activity in this model), ZX (expressed rNanA and rNanB in *E. coli* and purified the proteins), MR (confirmed the NA activity as well as its inhibition in HA assays), JK (computational modeling), UG and JR (isolation and characterization of katsumadain A), AK (isolation and characterization of Jena/8178), MS (MDCK cell studies) performed research and analyzed the data; WP, JR, AS, and MS contributed reagents/analytic tools; EW, ZX, MR, JK, AK, UG, JR, and SM analyzed data; and all authors wrote and approved the submitted version of the paper.

## Conflict of Interest Statement

The authors declare that the research was conducted in the absence of any commercial or financial relationships that could be construed as a potential conflict of interest.
