# Supplementary material for: Dual Acting Neuraminidase Inhibitors Open New Opportunities to Disrupt the Lethal Synergism between Streptococcus pneumoniae and Influenza Virus
Source: Front Microbiol. 2016 Mar 21;7:357. doi: 10.3389/fmicb.2016.00357 (PMC4800182; doi:10.3389/fmicb.2016.00357)
Supplement: Supplementary file 1 [file Data_Sheet_1.DOCX]

Supplementary Material

Dual-acting neuraminidase inhibitors open new opportunities to disrupt the lethal synergism between *Streptococcus pneumoniae* and influenza virus

Elisabeth Walther, Zhongli Xu, Martina Richter, Johannes Kirchmair, Ulrike Grienke, Judith Maria Rollinger, Andi Krumbholz, Andreas Sauerbrei, Hans Peter Saluz, Wolfgang Pfister, and Michaela Schmidtke^*^

*** Correspondence:** Michaela Schmidtke: [michaela.schmidtke@med.uni-jena.de](mailto:michaela.schmidtke@med.uni-jena.de)

# Supplementary Figures and Tables

## Supplementary Figures





**Supplementary Figure 1.** 4-MU standard curve.

**

**

**Supplementary Figure 2.** Cleavage activity of NanA and NanB. Michaelis Menten curves of NanA (squares) and NanB (dots) at pH 6.5. Both NAs were diluted 1:10,000 and incubated for 5 minutes with different substrate (MUNANA) concentrations. Kinetics was conducted three times. Mean and standard deviations were used to generate the graph.


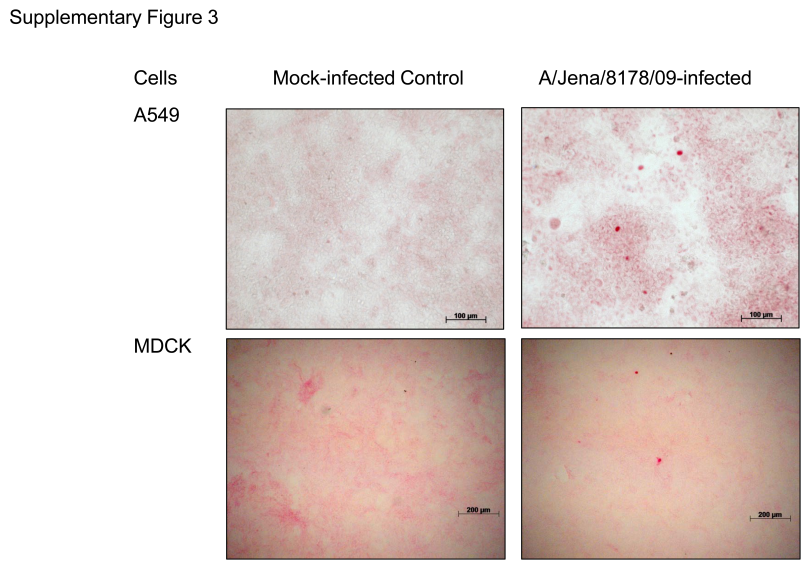


**Supplementary Figure 3.** Confirmation of A(H1N1)pdm09 strain A/Jena/8178/09 (Jena/8178)-infected A549 and MDCK cells after infection at low multiplicity of infection. Viral nucleoprotein was detected by immunohistochemical staining (virus-infected cells are seen as red dots) at 6 or 4 h after infection with Jena/8178 with 0.1 TCID50/cell in MDCK and A549 cell, respectively. Uninfected cells are shown for comparison.


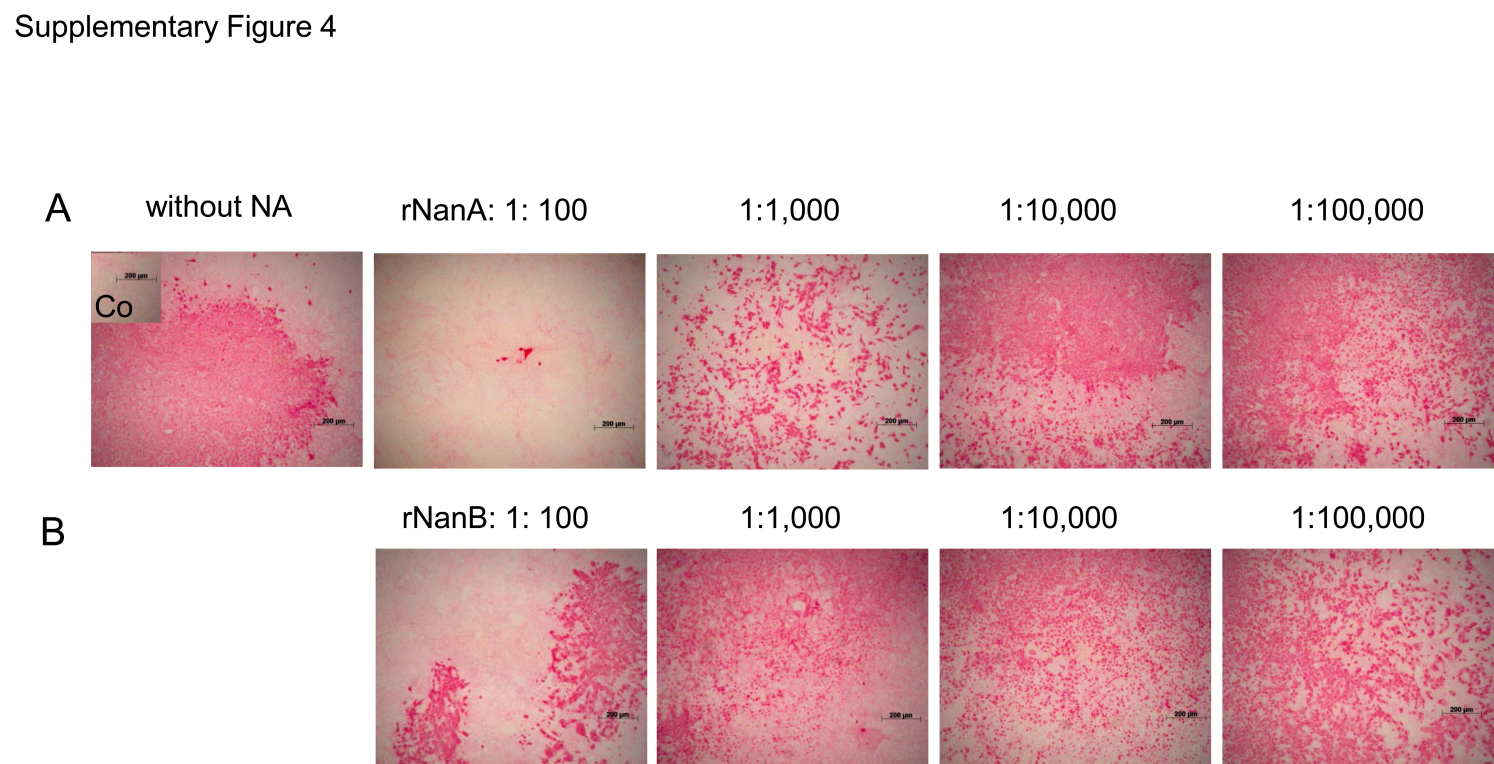


**Supplementary Figure 4.** Spread of A(H1N1)pdm09 strain A/Jena/8178/09 (Jena/8178) in absence and presence of different dilutions of recombinant NanA and NanB in MDCK cells. The effect of NanA (A) and NanB (B) on virus spread in MDCK cells was analyzed by immunohistochemical staining of viral nucleoprotein (shown in red) 48 h after infection with Jena/8178 at MOI of 0.1 TCID_50_/cell.


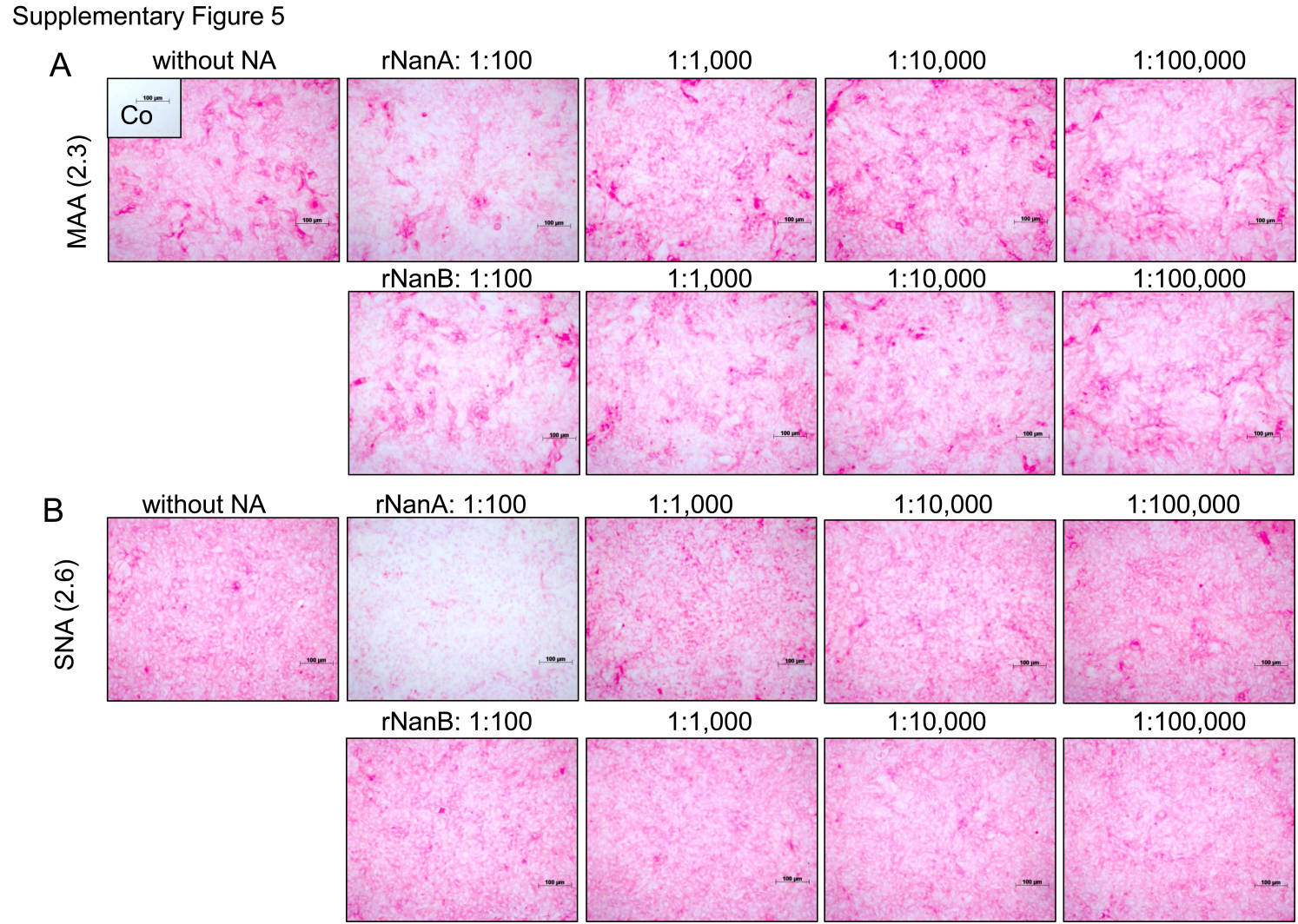


## Supplementary Figure 5. Influence of recombinant NanA and NanB on expression of sialic acids on the surface of MDCK cells. Cells were treated with different neuraminidase dilutions for 48 h. The lectins MAA and SNA were used to detect SAα2-3Gal and SAα2-6Gal, respectively, by immunohistochemical staining. Control (Co) were stained without using lectins.

## Supplementary Tables

**Supplementary Table 1.** Inhibition of the Influenza Virus A/Jena/8178/09 Neuraminidase by Neuraminidase Inhibitors (NAIs)

| **NAI** | **Inhibitory Concentration of NAIs in µM** |
| --- | --- |
|  | **FL Assay ^a^** |
| **Oseltamivir** | 0.003 ± 0.001 |
| **Zanamivir** | 0.005 ± 0.000 |
| **DANA** | 3.6 ± 0.4 |
| **Katsumadain A** | not evaluable ^b^ |
| **Artocarpin** | 41.8 ± 10.7 |

^a^ Mean 50% inhibitory concentration (IC_50_) and standard deviation determined in FL assay in at least three independent assays. The maximum tested concentration was 100 μM.

^b^ Not evaluable due to self-fluorescence of the compound.

**Supplementary Table 2.** Effect of Recombinant NanA (rNanA) and NanB (rNanB) on Inhibition of Virus Yield by Neuraminidase Inhibitors (NAIs)

| **NAI** | **Concentration**  **(µM)** | **Percentage of Virus Yield after NAI Treatment ^a^** | | |
| --- | --- | --- | --- | --- |
|  |  | **without NA** | **with rNanA** | **with rNanB** |
| **Oseltamivir** | 1 | 1.5 ± 1.1 | 3.2 ± 1.4 | 0.5 ± 0.2 |
|  | 0.1 | 16.7 ± 11.0 | 67.8 ± 24.8 | 4.5 ± 0.2 |
| **Zanamivir** | 1 | 1.4 ± 1.0 | 86.5 ± 46.2 | 0.7 ± 0.3 |
|  | 0.1 | 4.4 ± 1.5 | 109.0 ± 35.5 | 6.2 ± 2.9 |
| **DANA** | 50 | 34.8 ± 17.4 | 13.5 ± 8.3 | 27.4 ± 1.0 |
|  | 10 | 78.9 ± 36.1 | 81.1 ± 27.9 | not tested |
| **Katsumadain A** | 20 | 29.0 ± 15.1 | 28.1 ± 13.7 | 21.0 ± 4.1 |
|  | 10 | 54.9 ± 25.7 | 67.3 ± 6.2 | not tested |
| **Artocarpin** | 20 | 84.8 ± 15.7 | 44.3 ± 11.0 | 77.1 ± 37.8 |
|  | 10 | 70.2 ± 10.5 | 67.3 ± 25.5 | not tested |

^a^ The plaque titer of untreated control (influenzavirus A/Jena/8178/09) reached in A549 cells (48 h after infection) in the absence and presence of rNanA or rNanB was set as 100% virus yield. It was used to calculate the percentage of virus yield after NAI treatment. Mean and standard deviations were calculated of at least three assays with two replicates.
